# The Quantification of Radiation Damage in Orthophosphates Using Confocal μ-Luminescence Spectroscopy of Nd^3+^

**DOI:** 10.3389/fchem.2019.00013

**Published:** 2019-02-05

**Authors:** Christoph Lenz, Gordon Thorogood, Robert Aughterson, Mihail Ionescu, Daniel J. Gregg, Joel Davis, Gregory R. Lumpkin

**Affiliations:** ^1^Australian Nuclear Science and Technology Organisation, Sydney, NSW, Australia; ^2^Institut für Mineralogie und Kristallographie, Universität Wien, Vienna, Austria

**Keywords:** xenotime YPO_4_, monazite LaPO_4_, nuclear waste forms, rare-earth elements REE, luminescence spectroscopy, grazing-incidence X-ray diffraction, amorphous fraction, structural disorder

## Abstract

In this study, we present a new concept based on the steady-state, laser-induced photoluminescence of Nd^3+^, which aims at a direct determination of the amorphous fraction *f*_a_ in monazite- and xenotime-type orthophosphates on a micrometer scale. Polycrystalline, cold-pressed, sintered LaPO_4_, and YPO_4_ ceramics were exposed to quadruple Au-ion irradiation with ion energies 35 MeV (50% of the respective total fluence), 22 MeV (21%), 14 MeV (16%), and 7 MeV (13%). Total irradiation fluences were varied in the range 1.6 × 10^13^–6.5 × 10^13^ ions/cm^2^. Ion-irradiation resulted in amorphization and damage accumulation unto a depth of ~5 μm below the irradiated surfaces. The amorphous fraction created was quantified by means of surface-sensitive grazing-incidence X-ray diffraction and photoluminescence spectroscopy using state-of-the-art confocal spectrometers with spatial resolution in the μm range. Monazite-type LaPO_4_ was found to be more susceptible to ion-irradiation induced damage accumulation than xenotime-type YPO_4_. Transmission electron microscopy of lamella cut from irradiated surfaces with the focused-ion beam technique confirmed damage depth-profiles with those obtained from PL hyperspectral mapping. Potential analytical advantages that arise from an improved characterization and quantification of radiation damage (i.e., *f*_a_) on the μm-scale are discussed.

## Introduction

Orthophosphates of the REE^3+^[PO_4_]^−3^ group (with REE = Rare-earth elements Sc,Y + Ln; Ln = lanthanides La to Lu) have gained much attention in the past few decades from the nuclear materials science community. Their potential use as inert matrix fuel or waste-form material to immobilize hazardous actinides (e.g., U, Th, Pu, Np, Cm, Am) and fission products from high-level radioactive (HLW) wastes have been proposed in various studies (e.g., Boatner et al., 1980; Ewing and Wang, 2002; Lumpkin, 2006; Omel'yanenko et al., 2007; Weber et al., 2009; Burakov et al., 2011; Neumeier et al., 2017; Schlenz et al., 2018). The high structural flexibility of these compounds is one of their major advantages with respect to the latter purpose (Dacheux et al., 2013). At ambient conditions and depending on the radius of the REE cation, endmembers of this phosphate group either crystallize in the monoclinic monazite-structure type (La to Gd; space-group P2_1_/n) with 9-fold, or the tetragonal zircon-type structure (Gd to Lu, including Sc and Y; space-group I4_1_/amd) with 8-fold cationic coordination, whereas Gd-, Tb-, Dy-, and Ho-endmembers have been synthesized with both structure types (Ni et al., 1995; Kolitsch and Holtstam, 2004; Clavier et al., 2011). Orthophosphates of both structure types, however, have been reported to show a wide miscibility among their endmembers and the monazite structure has the ability to accommodate large amounts of the trivalent actinides Pu, Am, and Cm (Clavier et al., 2011 and references therein). The trivalent neutron absorber Gd can be accommodated easily in the crystal structure to control the criticality of radioactive chain-reactions at high actinide loadings. Heterovalent substitutions by mono-, di-, and tetravalent elements on cation sites are possible via vacancy, double or coupled charge-balanced substitutions. The latter substitution mechanisms are of major importance to accommodate Th^4+^, U^4+^, and other minor tetravalent actinides in the crystal structure of the orthophosphates (Clavier et al., 2011; Schlenz et al., 2018). Leaching experiments in static as well as dynamic experiment setups, performed to assess the ability of potential nuclear waste-form materials to retain structurally incorporated actinides upon leaching, reveal that orthophosphates have comparably low dissolution rates in hydrous and acidic media (Boatner and Sales, 1988; Tropper et al., 2011; Arinicheva et al., 2018; Gausse et al., 2018). A crucial factor that is generally considered to determine the long-term integrity of waste-form materials is their resistance against damage as caused by (self-)irradiation. The accumulation of radiation-induced damage in crystalline material is usually accompanied with reduced chemical durability and physical integrity, swelling and crack formation (Ojovan et al., 2018 and references therein). The interest in monazite-type compounds to be used as radiation-resistant fuel or waste-form material was substantially supported from findings of their natural analogs. The mineral monazite-(Ce) typically shows only moderate radiation damage accumulation, but to the best of our knowledge never has been found in an amorphous structural state. This is despite containing significant Th and U concentrations, which should have caused substantial accumulation of α-decay-induced radiation damage over geological periods of time (e.g., Boatner and Sales, 1988; Seydoux-Guillaume et al., 2004; Ruschel et al., 2012; Nasdala et al., 2018). The enhanced “radiation tolerance” in comparison to other accessory minerals like zircon (ZrSiO_4_), which is frequently found to be strongly damaged or metamict, have been attributed to self-annealing at comparably low temperatures. An effective accumulation of radiation damage is largely controlled by temperature-dependent kinetic effects (e.g., point-defect annealing, epitaxial recrystallization at crystalline-amorphous interfaces, recrystallization by nucleation) that may lead to simultaneous or post-damage recovery of the orthophosphates crystallinity (e.g., Nasdala et al., 2013). In comparison to zircon, exceptionally low critical amorphization temperatures T_c_ of REEPO_4_ orthophosphates have been identified by *in-situ* TEM (transition electron microscopy) ion-irradiation experiments (Meldrum et al., 1997a, 1998). Monazite-type phosphates have been found to have lower T_c_ (i.e., are more efficiently annealed) than phosphates with xenotime structure. Within the respective orthophosphate groups, T_c_ decreases systematically with increasing cationic radius (i.e., decreasing atomic mass) to be as low as 60°C for monazite-(La) (Meldrum et al., 1997a). Notably, Helean et al. (2004) found a linear positive correlation of T_c_ with the formation enthalpies ΔH_f−ox_ of the orthophosphate members, respectively. Thus, within the orthophosphate group, monazite-type LaPO_4_ is most thermodynamically stable with respect to its oxides (ΔH_f−ox_ ~-350 kJ/mol) and most efficient to anneal (*T*_c_ = 60°C), whereas xenotime-type LuPO_4_ is the least stable member (ΔH_f−ox_ ~-260 kJ/mol) and is annealed most inefficiently (*T*_c_ = 300°C). The annealing behavior of zircon fits well into the latter correlation. A comparably high T_c_ (1065°C; Meldrum et al., 1998) and a much lower thermodynamic stability (ΔH_f−ox_ = −28 kJ/mol; Ellison and Navrotsky, 1992) have been reported for zircon. The thermodynamic driving force to anneal radiation damage in zircon, hence, is much lower in comparison to the orthophosphates and may explain that annealing effects are more effective in the orthophosphates at lower temperatures. In addition to annealing effects as caused by elevated temperatures, α-particle induced annealing has been discussed recently to explain the absence of severe radiation damage of actinide-bearing orthophosphates stored at ambient (geo-)thermal conditions (Deschanels et al., 2014; Seydoux-Guillaume et al., 2018). For instance, single-phase ^238^Pu-doped La-monazite (Burakov et al., 2004; Deschanels et al., 2014; Shiryaev et al., 2016; Zubekhina and Burakov, 2016) or ^244^Cm-doped Lu-xenotime (Luo and Liu, 2001) stored under ambient conditions remained crystalline even at very high self-irradiation doses. On the other hand, however, monazite-type ^241^AmPO_4_ (Deschanels et al., 2014) and ^238^PuPO_4_ (Burakov et al., 2004) underwent metamictization at lower or comparable doses.

Studies that aim at an improved understanding of complex radiation-damage accumulation processes substantially benefit from an accurate quantification of the radiation damage present in the material of interest (e.g., Weber, 2000; Lang et al., 2009; Thomé et al., 2012; Liu et al., 2016). However, a quantitative determination of the amorphous fraction, i.e., the fraction of the material that underwent severe structural degradation, and the analytical access to effects caused by damage accumulation is very limited on the micrometer scale. The interpretation of diffuse scattering (as caused by the presence of amorphous material) in TEM investigations typically remains of qualitative nature and reliable estimates of the amount of damage present based on XRD techniques are restricted to monophase, bulk materials (e.g., Ríos et al., 2000; Lang et al., 2009). Rutherford backscattering and channeling (RBS/C) experiments give detailed information on the amount of displaced atoms, but are restricted to the characterization of radiation-damage accumulation in ion-irradiation experiments as non-damaged, crystalline starting material is needed for a quantitative interpretation (e.g., Thomé et al., 2012). In recent years, confocal spectroscopic techniques that operate on the micrometer scale, including Raman (e.g., Nasdala et al., 1995, 2010; Geisler et al., 2001; Picot et al., 2008; Shimizu and Ogasawara, 2014; Wang et al., 2014; Marillo-Sialer et al., 2016; Švecová et al., 2016; Baughman et al., 2017; Váczi and Nasdala, 2017; Zietlow et al., 2017) and luminescence spectroscopy (Panczer et al., 2012; Nasdala et al., 2013, 2018; Lenz and Nasdala, 2015) are applied frequently to characterize and estimate the degree of irradiation damage in orthophosphates and other accessory minerals. The latter techniques are based upon a correlation of spectral changes with structural modifications (i.e., band width broadening of narrow peaks) associated with radiation damage accumulation. Their applicability for a quantitative manner, however, is limited and hampered by a lack of reference samples of known amorphous fraction *f*_a_ or α-dose. For reasons discussed above, natural orthophosphate minerals do not serve as reliable references because of the insufficient knowledge of the samples annealing history. The damage characterized at present state, hence, cannot be correlated with its theoretically calculated α-dose, which gives the number of α-decays of a certain concentration of Th and U within the geological time span since its formation. For zircon, this problem has been addressed and discussed extensively (Geisler et al., 2001; Nasdala et al., 2001, 2010; Palenik et al., 2003; Lenz and Nasdala, 2015).

In the present study, we address the question of whether the analytical advantages of confocal spectroscopy can be combined with the possibility to quantitatively determine the amorphous fraction of radiation-damaged orthophosphates directly from single confocal luminescence spectroscopic measurements. To test this, we systematically accumulated radiation damage in polycrystalline, monophase LaPO_4_ and YPO_4_ ceramics using heavy ion-irradiation (Au) with various energies and fluences. The amount of structural breakdown, i.e., the amorphous fraction *f*_a_ created in the surface region of the irradiated ceramics, was then quantified using grazing-incidence X-ray diffraction (GI-XRD) and compared with results from confocal laser-induced photoluminescence spectroscopy of Nd^3+^. The orthophosphates are well-known for its excellent luminescence properties and have been proposed variously as effective phosphor pigments (e.g., Ropp, 1968; Gavrilović et al., 2018) or laser material (e.g., Guillot-Noël et al., 2000). In this study, however, we took advantage of the REEs unique spectroscopic properties to serve as structural probes of their local crystallographic environment (e.g., Bünzli and Piguet, 2005; Mendoza et al., 2011; Lenz et al., 2013). Emissions of Nd^3+^ have been favored among other potential REE probes here because its ^4^F_3/2_ → ^4^I_9/2_ electronic transition in the near-infrared (NIR) spectral range between 10,600 and 11,800 cm^−1^ (830–940 nm) is effectively excited in orthophosphates by standard steady-state lasers (e.g., Ar^+^ −488 nm, 514 nm; YAG:Nd^3+^ −532 nm; or diode pumped solid-state IR −785 nm) even at very low Nd concentrations in the ppm range. Moreover, the Nd^3+^ (^4^F_3/2_ → ^4^I_9/2_) emission is typically characterized by a small number of individual sublevels that simplifies data reduction (i.e., fitting/deconvolution). Other competitive luminescence emissions of REE^3+^ in the respective spectral range are rare and hence do not interfere with interpretation of the Nd^3+^ spectral signal (Lenz et al., 2013; Chen and Stimets, 2014). Moreover, Nd is commonly applied as non-hazardous surrogate in studies of actinide crystal chemistry of waste-form materials. Because of the high radioactivity and toxicity of e.g., Am, Cm, that are difficult to handle in standard laboratories, surrogates of similar chemical properties and behavior are applied frequently (e.g., Loiseau et al., 2004; Neumeier et al., 2017).

## Materials and Methods

Monophase phosphate powders of LaPO_4_ and YPO_4_ were produced using a wet-chemistry route. Oxide precursors (La_2_O_3_ and Y_2_O_3_; Sigma-Aldrich 99.95%) were dissolved in hot nitric acid and La- and Y-phosphate precipitates form, respectively, while dropwise adding an aqueous solution of (NH_4_)_2_HPO_4_. The pH was carefully adjusted to 9–10 using NH_3_ (aq.) to complete phosphate precipitation. To produce 100 g YPO_4_ monophosphate powder, 61.4 g Y_2_O_3_ was dissolved in ~220 ml HNO_3_ (3M) and 71.8 g (NH_4_)_2_HPO_4_ dissolved in 150 ml de-ionized water was added. For preparation of LaPO_4_ phosphate, 69.6 g La_2_O_3_ was dissolved in ~170 ml HNO_3_ (3M) and 56.5 g (NH_4_)_2_HPO_4_ dissolved in 150 ml de-ionized water was added to produce approximately 100 g of LaPO_4_ precipitate. Note, that an excess of approx. 20% HNO_3_ was needed to completely dissolve the respective precursor REE_2_O_3_ oxide. Dried precipitates were crushed with agate mortars, calcined at 800°C for 3 h, and ground in a zirconia-bead ball mill repeatedly to remove phosphate hydrates (Bregiroux et al., 2006; Sujith et al., 2014). Phosphate powders were then uniaxial cold-pressed to pellets of 10 mm diameter (steal die with a load of ~200 MPa) and sintered in the furnace at 1,300°C for 3 days. Sintered YPO_4_ pellets were found to have a specific gravity of ~4.08 g/cm^3^ as obtained from weight measurements with a hydrostatic balance and applying Archimedes principle. The density obtained is about 95.3% of the theoretical density 4.28 g/cm^3^ given by Milligan et al. (1982). Accordingly, LaPO_4_ pellets were determined to have a density of 4.56 g/cm^3^, which is 89.8% of the calculated density of 5.08 g/cm3 (Ni et al., 1995). The surfaces of ceramic pellets were polished using a Struers Tegramin preparation system with diamond suspension of various particle sizes (5–3 μm for coarse and 0.25 μm for fine polishing). A final chemical-abrasion procedure with colloidal silica (typical abrasive size is ~0.05 μm) was applied to remove mechanically induced stress at the surface during mechanical polishing.

**Figure 1:**
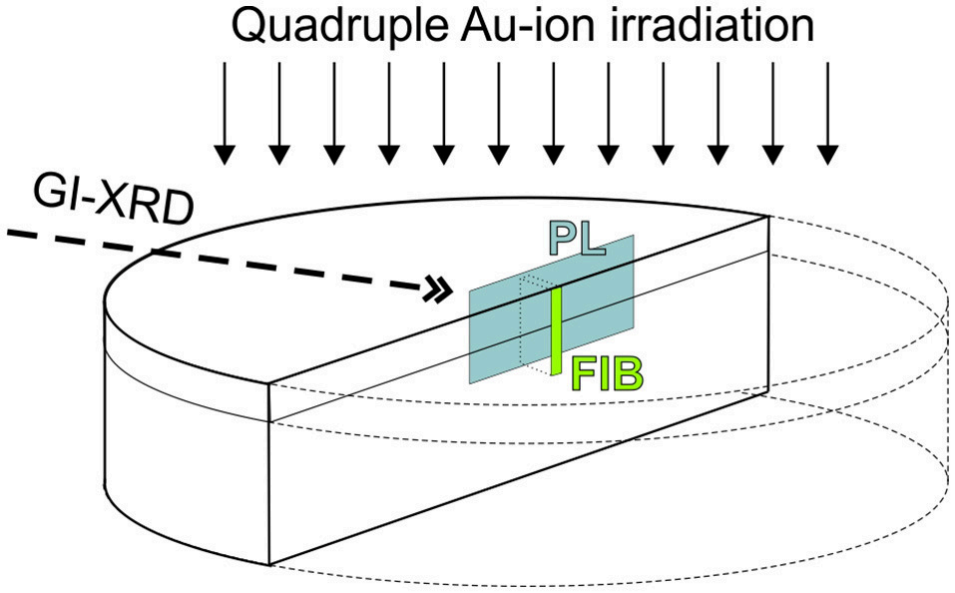
Simplified sketch (not to scale) that summarizes preparation and techniques as applied to orthophosphate ceramics in this study: (1) Polished ceramic pellets were irradiated with Au-ions sequentially with four different energies (see details in Figure 2 and discussion in the text); (2) grazing-incidence X-ray diffraction (GI-XRD) was applied to characterize ion-irradiation induced damage in the uppermost surface layer. (3) Pellets were embedded in epoxy and prepared to cross-sectional mounts; (4) confocal laser-induced Photoluminescence (PL) spectroscopy and hyperspectral mapping; and (5) focused ion-beam preparation of TEM foils.

**Figure 2:**
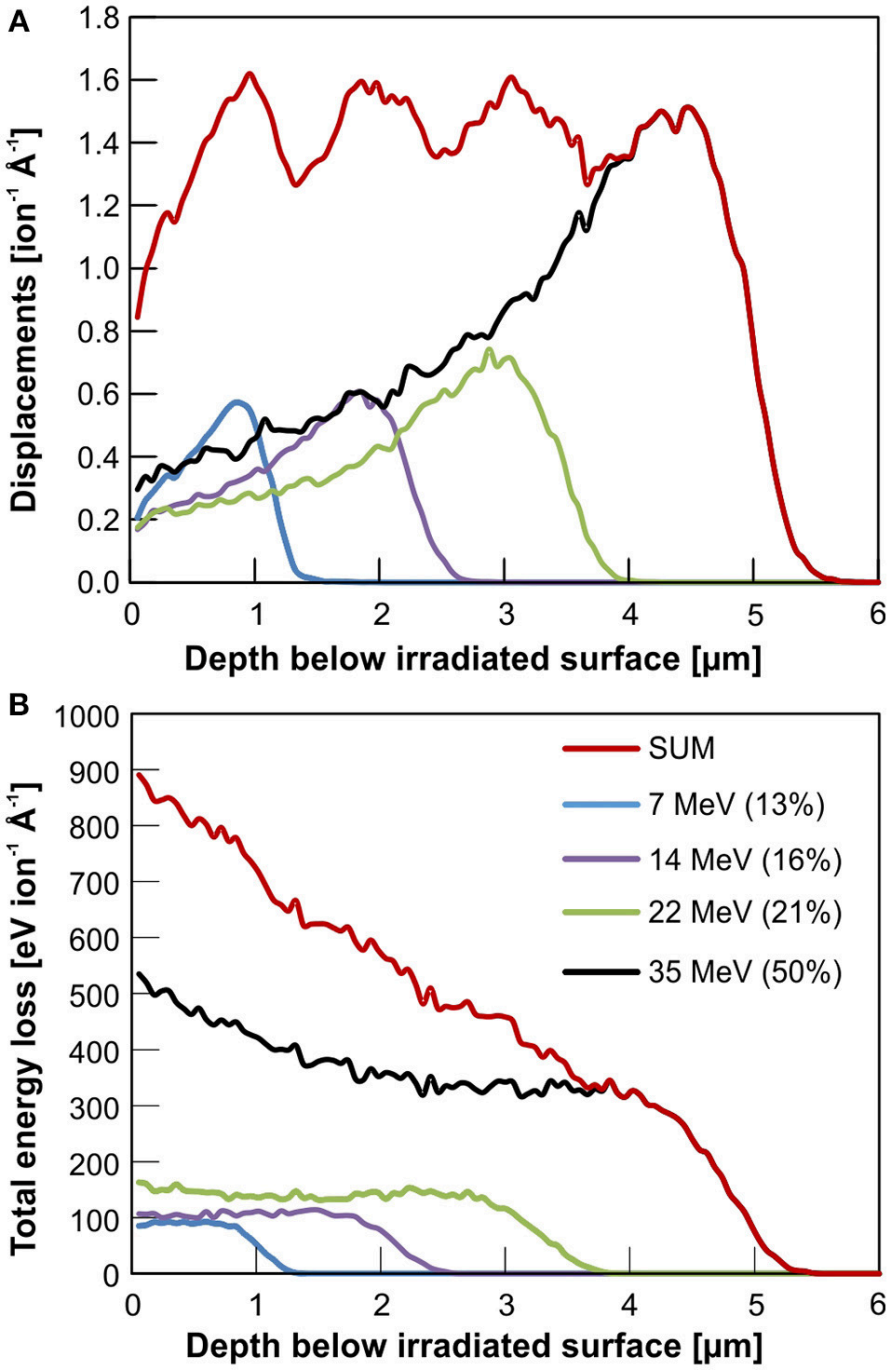
Monte Carlo simulation (SRIM-2013 code; Ziegler et al., 2010) of quadruple Au-ion irradiations of xenotime-type YPO_4_ using four different ion energies: 35, 22, 14, and 7 MeV. Relative fluences of 50, 21, 14, and 7% of the total fluence, have been chosen, respectively, to generate a homogeneous damage profile over a maximum irradiation depth of 5 μm as expected from the cumulative displacements predicted by SRIM **(A)**. The total energy loss of an incoming Au ion over the irradiation depth is shown in **(B)**. The latter comprise energy loss by nuclear (knock on) as well as electronic interaction (deceleration by coulomb interaction) with the target.

A graphical summary of analytical techniques and further preparation steps applied to LaPO_4_ and YPO_4_ ceramic pellets is given in Figure 1. Radiation damage was accumulated in the ceramics surface region utilizing irradiation of high energetic gold (Au) ions. Irradiation experiments were conducted using the 10 MV Tandem Van de Graaf, Australian National Tandem Research Accelerator (ANTARES) at ANSTO facility. Four different energies have been sequentially applied in the order highest to lowest energy (35, 22, 14, and 7 MeV). Picot et al. (2008), Nasdala et al. (2010), and Nasdala et al. (2018) have applied sequential triple ion-irradiations with the aim to create an extended, more homogeneous damage-depth profile in monazite-(Ce). We used the Monte Carlo simulation code SRIM-2013 (Stopping range of ions in matter; Ziegler et al., 2010) to calculate ion stopping-ranges and to pre-estimate damage accumulation as caused by the creation of displacements (full cascade mode). The rather high Au-ion energy of 35 MeV was chosen to produce a sufficiently large damage layer unto a depth of ~5 μm below the ceramic surface that is accessible to a broad range of analytical techniques. The four sequential Au-ion energies and their respective fluences were adjusted to generate a homogeneous damage profile over the irradiation depth as expected from cumulative displacements predicted by SRIM. Based on these calculations, each quadruple irradiation comprises 50% of the total fluence being from 35 MeV Au-ions, 21% from 22 MeV, 16% from 14 MeV and 13% from 7 MeV Au-ions (cp. Figure 2A). We exposed five LaPO_4_ and YPO_4_ ceramic pellets, each to five different total fluences (1.6 × 10^13^, 2.3 × 10^13^, 3.5 × 10^13^, 4.85 × 10^13^ and 6.5 × 10^13^ ions/cm^2^). Detailed fluences of each energy applied may be found in Table 1. Note that the ceramic pellets were irradiated at room temperature, i.e., cooling of samples during irradiation has not been applied. Samples were mounted on an aluminum plate with conductive tape, and the sample temperature was measured using K-type thermocouple. Monitored maximum temperatures did not exceed a maximum temperature of 45°C.

**Table 1:** Detailed Au-irradiation plan as applied to YPO_4_ and LaPO_4_ ceramics.

**Samples**		**Individual fluences of ion energies applied [ions/cm**^****2****^**]**				**Total fluence [ions/cm^**2**^]**
**YPO_**4**_**	**LaPO_**4**_**	**35 MeV (50%)**	**22 MeV (21%)**	**14 MeV (26%)**	**7 MeV (13%)**	**Sum (100%)**
d0	e0	0	0	0	0	0
d1	e1	8.00 × 10^12^	3.36 × 10^12^	2.56 × 10^12^	2.08 × 10^12^	1.6 × 10^13^
d2	e2	1.15 × 10^13^	4.83 × 10^12^	3.68 × 10^12^	2.99 × 10^12^	2.3 × 10^13^
d3	e3	1.75 × 10^13^	7.35 × 10^12^	5.60 × 10^12^	4.55 × 10^12^	3.50 × 10^13^
d4	e4	2.43 × 10^13^	1.02 × 10^13^	7.76 × 10^12^	6.31 × 10^12^	4.85 × 10^13^
d5	e5	3.25 × 10^13^	1.37 × 10^13^	1.04 × 10^13^	8.45 × 10^12^	6.50 × 10^13^

*Ceramic pellets were irradiated with Au-ions of four different energies in the order 35, 22, 17, and 7 MeV at fixed proportions, but with variable total fluences. A quadruple Au-irradiation was chosen to produce a homogeneous damage profile over a maximum irradiation depth of 5 μm as expected from the cumulative displacements predicted by SRIM (cp. Figure 2)*.

Irradiated ceramics and un-irradiated references were examined by GI-XRD performed on a Bruker D8 A25 fitted with a Cu source. The diffractometer was configured for parallel beam geometry using a Goebel mirror (deflection 0.812°) and a parallel plate collimator with an equatorial divergence of 0.2°. The lynxeye detector was configured to run in 0D mode to act as a point detector measuring in the 2θ range from 25 to 80°. Multiple runs with variable incident angles were performed to access and probe various depths below the surface. Details on incident angles and geometry specific X-ray attenuation lengths after Henke et al. (1993) are given in Table 2 and discussed in more detail below. Rietveld refinements were performed using Bruker AXS software package Topas Vers. 5.

**Table 2:** Detailed data of accumulated radiation damage in xenotime-type YPO_4_ ceramics of various depths below the irradiated surface as obtained from the two independent methods grazing-incidence X-Ray diffraction and laser-induced photoluminescence (Nd^3+^).

**YPO_**4**_ sample**	**Fluence [ions/cm^**2**^]**	**Amorphous fraction *f*_*a*_ from GI-XRD data**					Amorphous fraction *f*_*a*_ from PL data^*^			
		**Incident angle [°]**	**0.5**	**1.1**	**2.1**	**3.2**				
		**Attenuation length^§^ [μm]**	**0.25**	**0.50**	**1.00**	**1.50**	**Depth [μm]**	**1.5**	**2.5**	**5.0**
d0	0.00		0%	0%	0%	0%		0%	0%	0%
d1	1.60 × 10^13^		83%	78%	75%	69%		72%	66%	47%
d2	2.30 × 10^13^		92%	89%	87%	89%		88%	85%	68%
d3	3.50 × 10^13^		86%	81%	78%	72%		69%	60%	38%
d4	4.85 × 10^13^		93%	87%	85%	80%		78%	73%	54%
d5	6.50 × 10^13^		97%	95%	94%	90%		85%	80%	63%

§ *Approximate probing depths of X-rays at given incident angles and their attenuation lengths were calculated after Henke et al. (1993)*.

* *The amorphous fraction f_a_ is obtained from PL hyperspectral profiles across the irradiation-damaged surface. Multiple data points were (i) averaged unto distinct depths specified, and (ii) averaged among different profiles of various grains. See detailed discussion in the text. An error of ±5% is accepted to represent statistical variation*.

After XRD investigation, ceramic pellets were cut in half, and one of each half was embedded in epoxy and prepared to cross-sectional mounts and again carefully polished (see polishing procedure above). Steady-state, laser-induced photoluminescence spectroscopic measurements were performed using a Horiba LabRam Evolution HR800 spectrometer coupled to an Olympus BX80 optical microscope. A confocal hole in the analyzing beam path reduces the spatial resolution to 1 μm lateral and ~2–3 μm in depth using a 100 × objective. Here, we used a 532 nm frequency-double YAG:Nd^3+^ solid-sate laser with the beam path aligned through the microscope objective (quasi-backscattering configuration) to non-resonantly excite Nd^3+^ emissions that are most prominent in the orthophosphates (Lenz et al., 2013). Photoluminescence spectra in the NIR range were recorded using a grating with 600 lines/mm with a spectral resolution determined to be ~2 cm^−1^. Hyperspectral maps and transection profiles were obtained using a mechanic, software-controlled x-y table. Note, however, that although the lateral spatial resolution is limited to 1 μm, an over-stepping with step-widths of 0.2–0.5 μm was applied to maps and profiles to improve data and image quality.

Scanning electron microscopy (SEM) was performed using a Zeiss Ultra Plus Gemini equipped with an X-Max silicon drift detector Energy Dispersive X-Ray spectrometer (EDX). Specimen preparation for TEM was carried out using a Zeiss Auriga 60—focused ion beam (FIB) with Ga source. A 20 × 2 μm rectangular layer of protective platinum was deposited on the sample to protect the surface from the ion beam. Coarse milling of a trapezoidal shaped trench was performed on both sides of this platinum layer at 30 kV and 16 nA and then a rectangular shaped cut is done 1.5° off axis using a Ga beam with 30 kV and 2 nA to reduce the thickness of the lamella before lift-out. A “u” cut is then performed at shallow tilt angle in order to free the bottom and sides of the lamella. The lamella is lifted out *in situ* using an OmniProbe 200 nanomanipulator system and welded onto a TEM copper grid for further thinning and polishing inside the FIB. The polishing process involves positioning the lamella 0.5° off the axis of the ion beam on each side starting with a 30 kV and 1 nA, followed by a 30 kV and 120 pA ion probe thinning. Further fine thinning of the foils is done with 15 kV and 80 pA ion probe at 3° off axis to remove the damage created by the 30 kV gallium ions. Final polishing is done with a 5 kV 20 pA ion probe at 3° off axis and a long polish in deposition mode at 2 kV and 20 pA at 6° off axis milling to remove any further damage created by the higher energy gallium ions in previous steps. Thin TEM foils (with a final thickness <200 nm) of selected ceramics were prepared perpendicular to the irradiated surface of cross-sectioned ceramic samples. This was done to guarantee that complete irradiation-induced damage profiles of ~5 μm length are accessible to TEM within a single foil (cp. Figures 1, **6A**).

Transmission electron microscopy was carried out using a JEOL 2200FS operated at 200 kV. Specimen analysis of the cross-sectional damage depth profile was performed using bright-field and dark-field images. Lower magnification images were collected using a Gatan Orius SC200 D camera, whilst higher resolution images were collected on a Gatan UltraScan 1000. Selected area electron diffraction patterns (SADP) were collected using an aperture of approximately 600 nm diameter. Multiple SADPs were collected along the samples damage profile from the irradiated surface to the crystalline sub-surface in 0.5 μm steps. The Gatan Microscopy software-package DigitalMicrograph was used for image and diffraction pattern analysis.

## Results and Discussion

As revealed by optical microscopy (OM) and SEM investigation, sintering of LaPO_4_ and YPO_4_ cold-pressed pellets at 1,300°C for 3 days caused effective compaction and considerable grain growth with grain dimension of 1–10 μm and even larger grains up to 20 μm in case of YPO_4_ (cp. OM images in **Figure 7**). The phase structure was confirmed using GI-XRD. Rietveld refinement of XRD patterns revealed lattice constants in accordance to values reported in literature (YPO4: a = 6.88 Å, c = 6.01Å, cp. Milligan et al., 1982; LaPO4: a = 6.83 Å, b = 7.07, c = 6.50, β = 103.2°, cp. Ni et al., 1995). Pores appear occasionally in between grain boundaries that cause ceramics to have a lower density in comparison to theoretical values (theoretical calculated porosity of 4.7% in YPO_4_ and 10.2% in LaPO_4_ ceramics; see materials description in section Methods and Materials). A secondary, very minor phase was observed in YPO_4_ ceramics filling pendentives of large-grains. This phase, however, was found to be rich in Zr, contains no P (EDX) and show brighter contrast in SEM and reflectance in OM. We interpret this phase to be zirconia as introduced by milling with zirconia beads during preparation process. A minor XRD reflection at 2θ ~30° in XRD patterns of YPO_4_ ceramics (Figure 2A) may arise from this phase which was potentially formed during sintering by reaction of ZrO_2_ with omnipresent Y to tetragonal Y-stabilized zirconia (Y-TZP; Strasberg et al., 2014).

**Figure 3:**
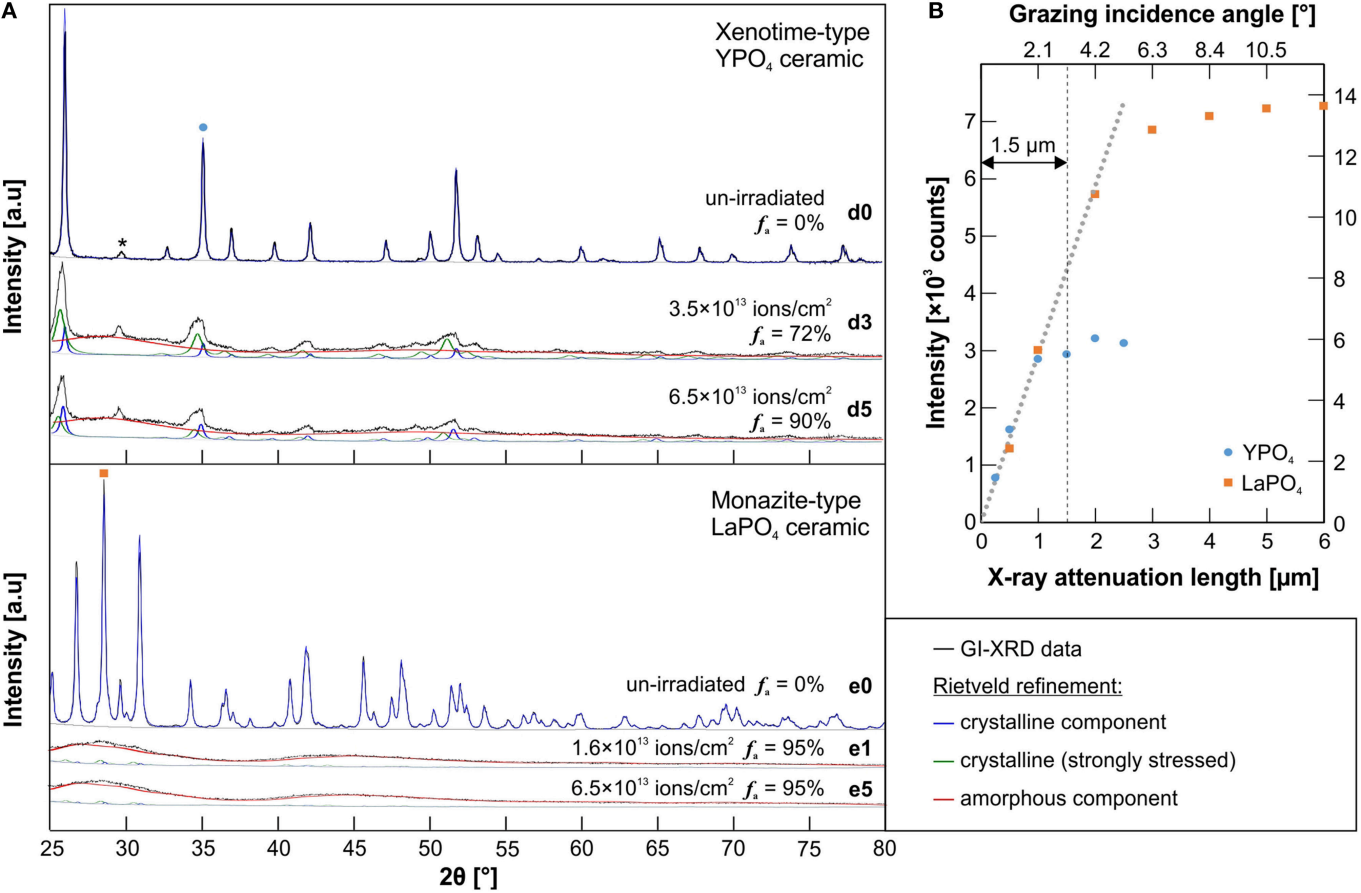
**(A)** A graphical illustration of the Rietveld refinement applied to grazing-incident X-Ray diffraction patterns of un-irradiated and irradiated xenotime-type YPO_4_ and monazite-type LaPO_4_ ceramics (cp. Table 1). The glancing angle was set to 3.2° to probe a thin surface layer unto a depth of ~1.5 μm. In YPO_4_ patterns, a minor diffraction peak at 2θ ~ 30° (marked with an asterisk) is attributed to a secondary phase (Y-stabilized tetragonal zirconia; Strasberg et al., 2014). To quantify the amorphous fraction *f*_a_, a refinement with three model phases have been applied: a fully crystalline component as observed from the un-irradiated sample (blue); a strongly stressed but still crystalline component (green); and an amorphous component refined with free lattice constants and very small grain-size factors (red). **(B)** The intensity of representative reflections of un-irradiated YPO_4_ [(211) at 2θ ≈ 35°, left-hand y-axis] and LaPO_4_ ceramic [(120) at 2θ = 28.6°, right hand y-axis] in dependence of various glancing angles that correspond to certain X-ray attenuation lengths calculated after Henke et al. (1993) (cp. Table 1). GI-XRD intensities of un-irradiated references samples saturate significantly above attenuation lengths >2 μm. An accurate probing depth of this method was accepted to be until ~1.5 μm below the surface for both phases.

To establish the limits of the GI-XRD probing depth, un-irradiated reference samples of both, LaPO_4_ and YPO_4_ ceramics were examined in detail. Diffraction patterns obtained by using various grazing angles, which correspond to certain calculated X-ray attenuation lengths (Henke et al., 1993), were compared. In Figure 3B, the intensity of major reflection peaks of both ceramic references are plotted against the X-ray attenuation lengths as calculated from the respective incident angles. The higher the incident angle of the X-ray beam is set, the deeper it may penetrate into the material. In practice, the probing depth is limited effectively by the materials specific X-ray absorption, geometrical constraints, and measurement specific analytical conditions (e.g., type of X-ray source and respective energy). The incident angle above which the intensity of diffraction peaks does not increase any further is interpreted to reflect the maximum probing depth. Intensities of un-irradiated references saturate significantly at attenuation lengths >2 μm (Figure 3B). Hence, a probing volume that reaches a depth unto ~1.5 μm was accepted to most reliably be characterized with GI-XRD. This corresponds to an instrument set-up with the X-ray beam set to an incident angle of max. 3.2° for both phases.

The effect of quadruple ion-irradiation on the structural state of irradiated xenotime-type YPO_4_ and LaPO_4_ ceramics and its GI-XRD patterns is demonstrated in Figure 3A. Irradiations of Au onto YPO_4_ with total fluences of 3.5 × 10^13^ (d3) and 6.5 × 10^13^ ions/cm^2^ (d5) caused diffraction peaks to significantly decrease and broaden, together with broad humps that appear across the entire diffraction pattern in the background. The latter contribution to the diffraction pattern is considered to arise from diffuse scattering of X-rays from the surface layer that is altered by ion-irradiation, lost long-range order, and hence, represents the amorphous component in the analyzed volume fraction. Note, however, that remnant YPO_4_ diffraction peaks broadened asymmetrically with a shift to lower 2θ, and hence, indicate increasing lattice planes *d* and swelling of the unit cell. This is a common effect accompanied with radiation damage accumulation in ceramic materials (e.g., Trachenko et al., 2002; Deschanels et al., 2014). In contrast to YPO_4_, GI-XRD patterns of all irradiated LaPO_4_ ceramics are characterized by the presence of broad humps only. Almost complete amorphization of the irradiated surface was detected even at the lowest irradiation fluence applied (1.6 × 10^13^ ions/cm^2^; see LaPO_4_ sample e1 in Figure 3A).

To properly quantify the degree of amorphization in the irradiated surface layer, we applied a Rietveld refinement using three chemically identical, but structurally different model phases that are deduced from the reference structure model and considered to persist simultaneously in the ion-irradiated ceramics: (i) fully crystalline remnants that are identical to the un-irradiated references, (ii) a strongly stressed and disrupted, but still crystalline component that is modeled with carefully adjusted lattice constants and grain size factors to account for substantial peak broadening and shifting, and (iii) the amorphous component modeled with unrealistic, freely refined lattice constants and very low grain size factors to represent the broad humps that underlay remnant diffraction peaks. Note, however, that a general background correction applied to GI-XRD patterns of irradiated ceramics was adopted from the un-irradiated reference and assumed to be comparable due to the very same analytical measurement conditions. Graphical representations of refinement results are given in Figure 3A. The amorphous fraction *f*_a_ of the irradiated YPO_4_ ceramics as quantified from GI-XRD Rietveld refinement is denoted and further summarized in Table 2 for various incident angles (i.e., probing depths). All five irradiated monazite-type LaPO_4_ pellets are characterized by strong accumulation of radiation damage in the surface layer. All of the irradiated LaPO_4_ ceramics gave similar patterns with broad humps and almost no remnant diffraction peaks. An equivalent Rietveld refinement as applied to YPO_4_, yielded a very high amorphous fraction of 95 ± 5% (see again Figure 3A).

**Figure 4:**
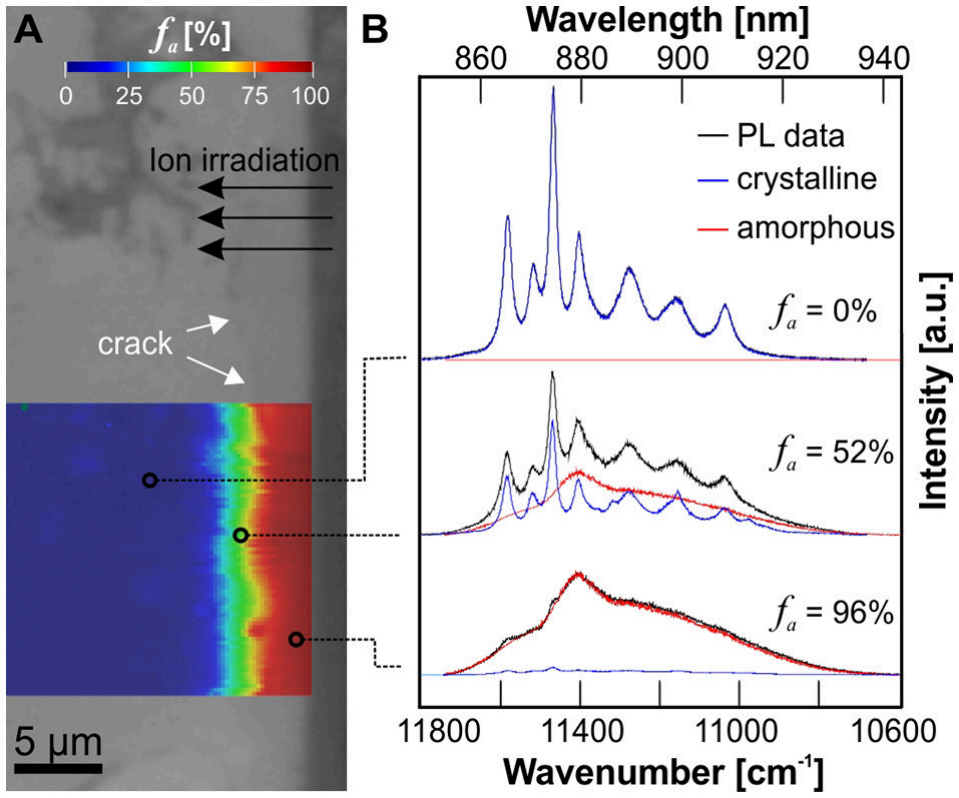
**(A)** Optical micrograph image of a cross-sectioned LaPO_4_ ceramic (e1, irradiated with 1.5 × 10^13^ ions/cm^2^) superimposed by a PL hyperspectral map with the amorphous fraction *f*_*a*_ given color-coded. **(B)** Representative PL spectra of the Nd^3+^ emission (^4^F_3/2_ → ^4^I_9/2_) obtained from various depths below the irradiated surface illustrating the deconvolution procedure used to determine *f*_*a*_ from PL data. The red component represents a spectrum of the very same Nd^3+^ emission from a completely amorphized reference sample. Note, that the lowest irradiation fluence applied in this study was sufficient to produce an almost completely amorphized surface layer in LaPO_4_.

**Figure 5:**
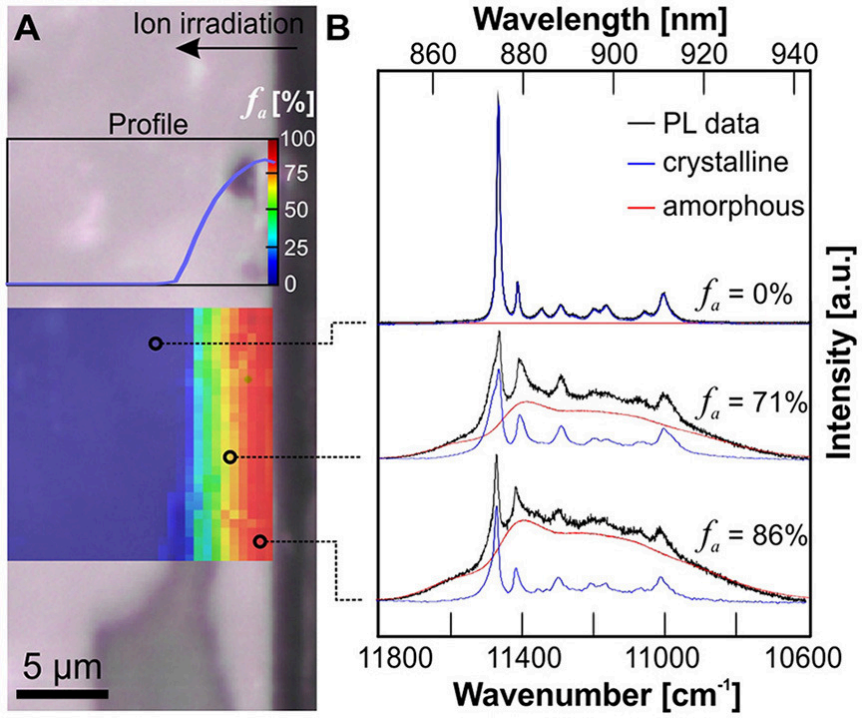
**(A)** Optical micrograph image of a cross-sectioned YPO_4_ ceramic (d5, irradiated with 6.5 × 10^13^ ions/cm^2^) superimposed by a PL hyperspectral map with the amorphous fraction *f*_*a*_ given color-coded. **(B)** Representative PL spectra of the Nd^3+^ emission obtained from various depths below the irradiated surface illustrating the deconvolution procedure used to determine *f*_*a*_ from PL data. The red component represents a PL spectrum of the very same Nd^3+^ emission from a completely amorphized reference sample. The accumulated radiation-damage shows a non-uniform profile with *f*_*a*_ decreasing from surface to a depth of ca. 5 μm.

**Figure 6:**
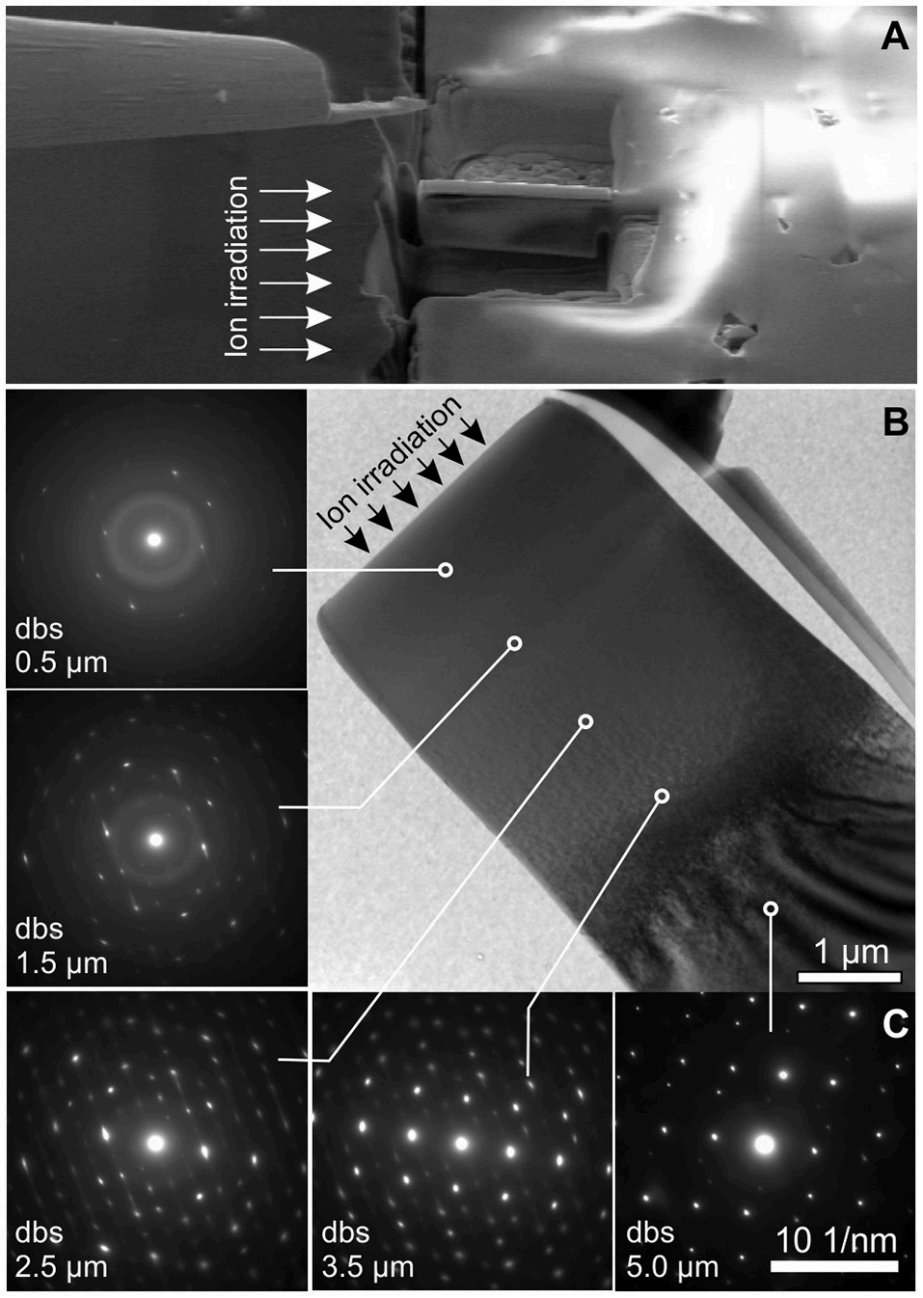
**(A)** SEM image illustrating the focused-ion beam (FIB) preparation of a TEM-foil obtained from the cross-sectioned irradiated surface of YPO_4_ ceramic sample d5 (the direction of the ion-irradiation indicated by arrows). **(B)** TEM bright-field image of the FIB-prepared foil with selected-area electron-diffraction (SAED) patterns from various depths below the irradiated surface (dbs). Diffuse scattering in SAED patterns decrease with depth, indicating a decreasing fraction of amorphous YPO_4_ from the surface to a depth of ~5 μm.

**Figure 7:**
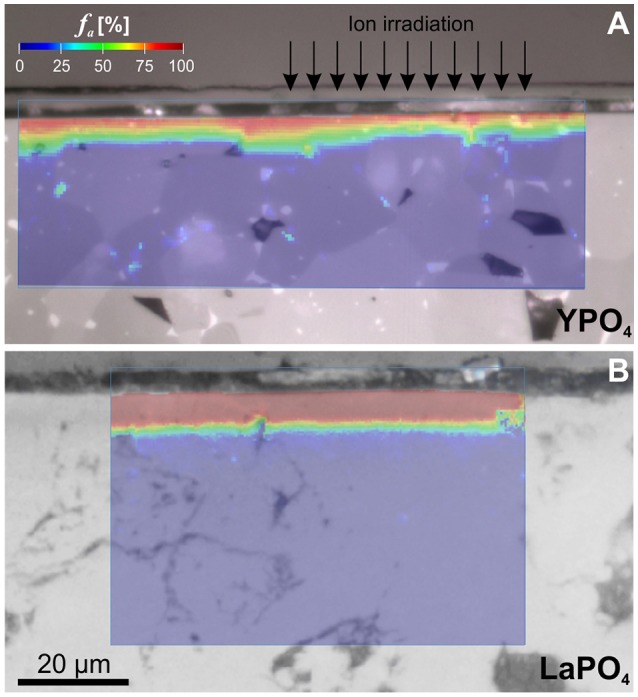
Optical micrograph images (reflection) of cross-sectioned orthophosphates ceramics superimposed by PL hyperspectral maps with the amorphous fraction *f*_*a*_ given color-coded (determined as illustrated in Figures 4, 5). **(A)** YPO_4_ ceramic d4 irradiated with a total fluence of 4.85 × 10^13^ ions/cm^2^. Note, that the dimension of the irradiated surface layer is not homogeneous across various grains. The irradiation depths vary notably with crystal orientation relative to surface-directed ion-irradiation (indicated by arrows). **(B)** LaPO_4_ ceramic e1 irradiated with a total fluence of 1.5 × 10^13^ ions/cm^2^. The entire irradiated 5 μm thick surface layer was found to be almost completely amorphized at the lowest irradiation fluence applied.

Laser-induced photoluminescence of Nd^3+^ (^4^F_3/2_ → ^4^I_9/2_) in monazite and xenotime-type orthophosphate ceramics was effectively excited with a 532 nm laser. Sufficient signal intensity in the spectral range 11,800–10,600 cm^−1^ (~ 830–940 nm) was obtained in high-confocal measurement mode that permits the probing volume to be reduced to a few μm3. Note that the orthophosphates were not doped with additional Nd during their preparation process. Trace impurities of Nd in the precursor Y and La-oxides were sufficient to obtain its very sensitive PL. The impact of heavy-ion irradiation-induced structural disorder on the emission of Nd^3+^ is exemplified in Figures 4, 5. They show hyperspectral images of the cross-sectional damage profile of LaPO_4_ pellet e1 (irradiated with 1.5 × 10^13^ ions/cm^2^), and YPO_4_ pellet d5 (irradiated with a total fluence of 6.5 × 10^13^ ions/cm^2^), respectively, in addition to representative single spectra from various depths across the damage profile. Note that crystal-field split sub-level bands are easily identified in spectra of the unaffected crystalline area of both materials. Those narrow bands are characteristic luminescence features that arise from REEs shielded electronic intra *f–f* transitions. Their number and positions are strongly dependent on the local cationic structural environment the Nd ions are incorporated in, and hence, are specific for indivuidual phases (cp. Nd emission from 9-fold cation-site in monazite vs. 8-fold site in xenotime-type ceramic in Figures 4, 5). Structural disorder is typically interpreted to result in inhomogeneous broadening of these PL bands that arise from statistical variation of the local cationic crystal field as induced by stress/strain and/or structural defects (Skinner and Moerner, 1996; Liu et al., 2000; Lenz et al., 2013). In addition to band-broadening, however, we observed that a seperate broad-band component emerges in Nd^3+^ emission spectra of irradiated orthosphosphates in dependence of the amount of radiation-damage accumulation which is most dominant close to the irradiated surface. This component is marked as red-hatched curve in spectra of Figures 4, 5 and represents a spectrum of the very same Nd^3+^ emission of completely amorphized reference samples, i.e., narrow bands from spectroscopic sub-levels are not observable and degenerated completely. That component in spectra of radiation-damaged xenotime- and monazite-type ceramics may be interpreted as emissions arising from Nd^3+^ ions in a completely “amorphized” cationic environment. The detected PL signal of individual spot measurements (of μm^3^-volume) are, hence, considered to be a superposition of emissions from a multitude of individual Nd ions in sites with different degree of structural integrity. We used the integrated intensity of the deconvoluted amorphous component in relation to the overall integrated intensity of the Nd^3+^ emission as a reasonable estimate of the amorphous fraction *f*_a_ present. The latter estimate is valid given the assumption that trace Nd ions are distributed statistically within the analyzed volume and all of them contribute similarily to the detected PL signal (i.e., the quantum conversion is the same and independent among different sites). Note, however, that the relation of these two components is in principle independent of the absolute intensity of the luminescence signal, which may strongly depend on (1) the luminescence scattering profile of the analyzed sample surface determined e.g., by the surface roughness, (2) the concentration of Nd ions present in the sample and its distribution, and on (3) further potential luminescence quenching effects (e.g., quenching by irradiation-induced defect centers that have been reported to cause a substantial decrease in luminescence intensity in zircon: for photo- and cathodoluminescence see Lenz and Nasdala, 2015; for ionoluminescence cp. Finch et al., 2004). We applied an automated deconvolution (i.e., a least-squares component fitting) to all individual spectra (i.e., pixels) of the hyperspectral maps obtained from the cross-sectional damage profiles with results given color-coded in Figures 4, 5. The irradiated surface area of LaPO_4_ ceramics is heavily damaged by Au-irradiation with an *f*_a_ estimated to be around 96% (Figure 4) that is consistent with f_a_ observed with GI-XRD. While the probing depth of GI-XRD is limited to max. 2 μm, confocal PL mapping of the cross-sectioned surface of LaPO_4_ ceramics revealed a damage profile that is characterized by a very high structural damage unto depths 4–4.5 μm, followed by a strong continous reduction of *f*_a_ unto the maximum irradiation depth at ~5 μm (cp. **Figure 7B**). Somewhat different trends of damage accumulation are found for irradiated YPO_4_ ceramics. Here, the maximum damage levels are limited to the surface region, are much lower than for LaPO_4_, and subsequently decrease appreciably unto a depth of 5 μm (see *f*_a_ profile in Figures 5A, 7A). Note that, similar to diffraction peaks in GI-XRD patterns, spectroscopic bands of the remnant crystalline fraction in YPO_4_ broadened assymetrically (see shoulder next to the main peak at 11,470 cm^−1^) which confirms the presence of a strongly altered and stressed, but still crystalline fraction. The latter inhomogeneous trend of damage accumulation in YPO_4_ was qualitatively confirmed using TEM with results summarized exemplarily in Figure 6 (sample d5; ion-irradiated with a total fluence of 6.5 × 10^13^ ions/cm^2^). Thin TEM foils were prepared from the irradiated surface area by FIB preparation so that the entire ion-irradiation induced damage profile is accessible to TEM (Figure 6A; cp. Figure 1). Diffuse scattering in selected-area electron-diffraction (SAED) patterns as obtained from various depths below the irradiated surface indicate a decreasing presence of amorphous material from surface to a depth of 5 μm. Contrasts in TEM bright-field (BF) images are homogeneous and SAED patterns are characterized by bright diffuse rings unto a depth of 1.5 μm. Contrast features in BF are increasingly apparent with increasing depth below the surface (dbs) and finally lattice fringes are clearly visible at the end of the damage profile, together with a loss of diffuse scattering in SAED patterns. Diffraction spots from crystalline components between 0.5 and 4 μm smear out notably in discrete crystallographic directions in reciprocal space. Continuous shifts of diffraction spots in SAED patterns are considered as TEM analog to effects observed with peaks in GI-XRD patterns that shift to lower 2θ (i.e., increasing lattice planes distances *d*), and again, interpreted to be a result from strongly stressed crystalline remnants in irradiated YPO_4_.

The accumulation of radiation damage in the YPO_4_ orthophosphate irradiated surface layer is high close to the surface and decreases with irradiation depth. This is confirmed by three independent methods as applied in this study (cp. *f*_a_ as obtained by GI-XRD in shallow dbs in Table 2; quantitative PL damage profiles in Figure 5; and qualitative TEM results discussed above, Figure 6). Obtained damage profiles clearly do not resemble expected theoretical, though qualitative, results of SRIM Monte-Carlo simulations that predict a rather homogeneous accumulation of displacements over an ion penetration depth of 5 μm (cp. Figure 2A). We consider two potential reasons that may explain deviations from the theoretical damage profile as observed in our irradiation experiments. Firstly, epitaxial, concurrent or post-irradiation annealing may emanate from the unaffected crystalline back into the stopping range of Au ions at the end of their trajectory. As briefly reviewed in the introduction, orthophosphates are known for their sensitivity to spontaneous annealing as induced by the subtle increase of temperature (Meldrum et al., 1997a), and dissemination of energy due to electronic excitation as observed from electron-beam induced nucleation and growth (Meldrum et al., 1997b) or electronic stopping as discussed for α-particle annealing (Deschanels et al., 2014; Seydoux-Guillaume et al., 2018). Progressive epitaxial recovery of crystallinity at the crystalline/amorphous interfaces has been reported e.g., from *in situ* TEM annealing experiments of ion-irradiated pyrochlores (Aughterson et al., 2018). Secondly, damage profiles on the basis of displacements predicted by SRIM may be insufficient to describe damage accumulation as caused by heavy ions in the MeV range as applied in our study. The amount of displacements calculated in SRIM simulations is deduced from the deposition of nuclear-stopping energy only. Amorphization that arises from electronic interaction of high-energy heavy ions with the bombarded material is not accounted for in SRIM calculations. Various experiments with heavy-ions in the MeV and GeV energy range that show a large electronic stopping regime (i.e., swift heavy ions) have demonstrated that the dissemination of electronic stopping energy through the interaction of high-energetic, charged ions with the coulomb-field of bombarded materials resulted in formation of amorphous ion tracks (e.g., Sales et al., 1992; Toulemonde et al., 2001; Lang et al., 2009; Liu et al., 2016). A comprehensive overview of models that explain ion-beam damage in the electronic stopping regime is given by Agulló-López et al. (2016). The impact of electronic energy loss of irradiated MeV ions on the accumulation of radiation damage as an explanation of damage profiles observed in our experiments is supported by SRIM calculations of ion energy-loss that includes both contributions, nuclear as well as electronic stopping (Figure 2B). The cumulative profile of energy loss, however, reproduce damage profiles obtained in this study more concisely.

**Figure 8:**
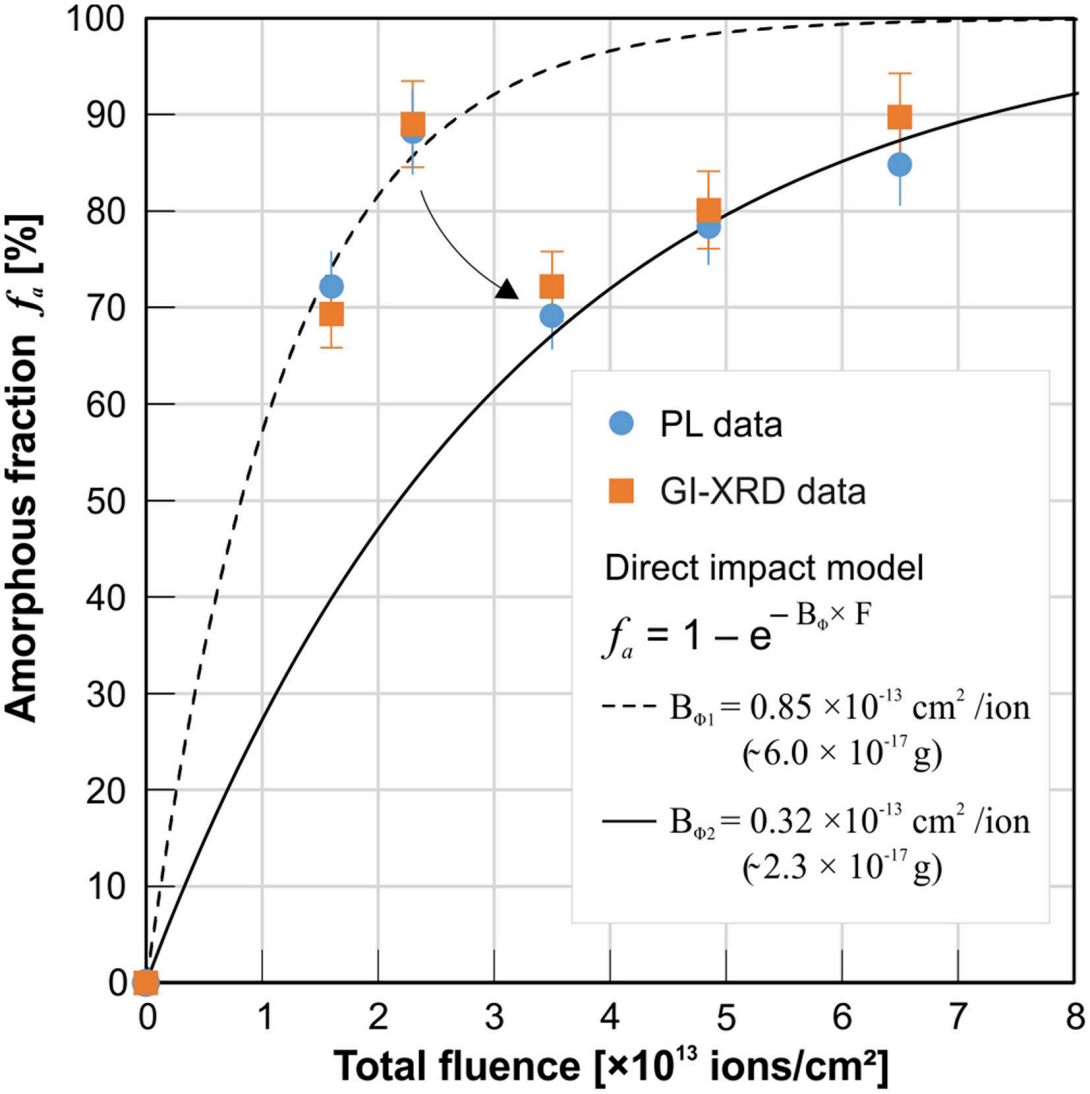
Summarized data from PL and GI-XRD measurements of heavy-ion (Au) irradiated YPO_4_ ceramics: The amorphous fraction *f*_a_ of the irradiated surface unto a depth of 1.5 μm as obtained from the two independent methods plotted against the applied total fluence (see details on irradiation energies used in Figure 1; see detailed data in Table 2). Both methods give comparable *f*_a_ values with respect to their error margins. Note, that radiation-damage does not accumulate continuously with increasing fluence. At high radiation damage levels as accumulated >2 × 10^13^ ions/cm^2^, a sudden set-back accompanied with a less effective accumulation behavior was registered (arrow).

Despite apparent discrepancies between damage profiles calculated by SRIM and those observed in our experiments, predictions on the ion-penetration trajectory-lengths were found to be reasonably accurate to a first approximation. Hyperspectral PL maps indicate damage accumulation to extend to a depth of ~5 μm in both, LaPO4 and YPO_4_ ceramics, as predicted from the stopping range of the maximum applied Au-ion irradiation with 35 MeV (cp. Figure 2). This is most apparent from LaPO_4_ ceramics that were entirely amorphized throughout the irradiated surface layer (Figure 7B). In YPO_4_ ceramics, however, we found small deviations in irradiation depths in dependence of the crystal orientation relative to the surface directed ion-beam (Figure 7A). Depths of damage accumulation as revealed by detailed PL hyperspectral images across multiple grains were found to deviate slightly between adjacent crystal grains. Also, grain boundaries and pores that phase out at the polished surface were found to promote damage accumulation in those areas. Variable radiation-damage depth-profiles across different grains complicate the comparability of quantitative estimates of the amorphous fraction *f*_a_ as obtained from the surface-sensitive, but bulk method GI-XRD and point analyses of confocal PL spectroscopy in the μm-range. To quantitatively compare results from PL depth profiles of *f*_a_ with those from GI-XRD, we (i) averaged *f*_a_ values obtained from PL depth profiles unto the maximum effective probing depth of GI-XRD at 1.5 μm, and further (ii) took the mean from multiple depth profiles of the same and/or different grains to account for statistical variation as introduced by effects discussed above. Latter results are summarized in Table 2 and plotted in Figure 8 together with *f*_a_ data obtained from GI-XRD. With their strongly deviating probing volumes taken into account, both techniques give very consistent estimates of the amorphous fraction *f*_a_ within their error margins of ±5%. Note, however, that both applied techniques reveal an extraordinary discontinuous damage-accumulation behavior of YPO_4_ with increasing Au-ion fluence (see Figure 8 again). The amount of radiation damage increases with the first two applied irradiation fluences (d1 = 1.6 × 10^13^ ions/cm^2^; d2 = 2.3 × 10^13^ ions/cm^2^) that result in a high *f*_a_ ~ 90% present in the uppermost irradiated layer. The accumulation of the amorphous fraction *f*_a_ follows the direct impact model as given in Figure 8 (with variables B_Φ_ = cross-section of amorphous ion tracks, and F = Au-ion fluence applied). A decrease of *f*_a_ was then recorded for xenotime-type YPO_4_ sample d3 irradiated with a slightly elevated fluence at 3.5 × 10^13^ ions/cm^2^ (see arrow in Figure 8). Fluences >3.5 × 10^13^ ions/cm^2^ cause the radiation damage to accumulate again, but with less efficiency as demonstrated by the regression model with a much lower ion-track cross-section B_Φ2_. The latter erratic damage-accumulation behavior accounts for a competitive process that prevents and counteracts further damage accumulation by MeV Au ion-irradiation at high *f*_a_. Potential epitaxial annealing effects as caused by the input of energy due to high electronic loss of MeV swift ions have been reported for orthophosphates (Rafiuddin and Grosvenor, 2015), and repeatedly for SiC (Benyagoub et al., 2006; Debelle et al., 2012; Backman et al., 2013). In this study, however, we cannot clarify conclusively if the impact of electronic energy loss is the most probable explanation for competitive annealing observed at elevated total ion-fluences in YPO_4_, because temperature-assisted annealing may occur as a result of sample heating at higher fluences (an increase of sample temperature to a maximum of 45°C have been recorded during irradiation experiments). To differentiate between annealing effects as caused by the impact of electronic energy loss of MeV heavy-ions and potential sample heating, we strongly suggest to apply effective sample cooling (e.g., liquid nitrogen) during ion-irradiation of the very temperature-sensitive orthophosphates.

## Conclusions and Implications

The luminescence of Nd^3+^ (^4^F_3/2_ → ^4^I_9/2_) in monazite-type and xenotime-type orthophosphates was identified as a promising structural probe that bears quantitative information on the degree of amorphization as induced by ion-irradiation. Careful investigation of PL spectra from heavy-ion irradiated ceramic surfaces revealed that the detected luminescence signal may be interpreted as a superposition of emissions from Nd ions situated in structurally different components in the damaged crystal structure. The latter comprise completely amorphous domains (with a structurally degenerated Nd-cation environment) that are created upon damage accumulation and persist next to unaffected or slightly affected, stressed crystalline remnants on a submicroscopic scale. We found, that the integrated area of the deconvoluted amorphous component in relation to the fully integrated luminescence signal gives a reliable estimate of the amorphous fraction *f*_a_ present in the probed sample volume. This approach was substantiated and confirmed by quantitative Rietveld refinements of GI-XRD patterns obtained from ion-irradiation damaged LaPO_4_ and YPO_4_ ceramics.

Laser-induced PL spectroscopy has a number of analytical advantages, that includes (1) the option to perform analyses non-destructively and without the need for special sample preparation, (2) the application as a remote technique e.g., through optical transparent windows if protection against hazardous samples is needed, and (3) the possibility to perform spot analyses using highly confocal spectrometers coupled to optical probe-heads or microscopes with the latter operating on the micrometer length-scale. The analytical flexibility to detect the Nd^3+^ (REE^3+^) luminescence in the orthophosphates opens up the opportunity to investigate their complex damage-accumulation behavior in more detail and give rise to an improved comparability to results obtained from very different samples or experiment setups, e.g., studies that require quantification of the amorphous fraction in natural mineral-analogs, self-irradiation damaged synthetic samples doped with fast decaying actinides such as ^238^Pu, ^241^Am, or ^244^Cm, in ion-irradiation experiments, or studies that involves minute *in-situ* monitoring of *f*_a_ in e.g., radiation-damage annealing studies under controlled conditions. Further, quantitative information of structural damage may be deduced from REE luminescence in advanced orthophosphate nuclear waste-forms that is not stimulated externally (e.g., PL: with optical lasers or UV lamps), but by inherent β- or γ-radiation from the radioactive decay of substituted actinides (radioluminescence). “Self-glowing” phosphate crystals that contain highly-active actinides and adjusted concentrations of luminescing REEs have been prepared successfully (Burakov et al., 2011). We, however, consider that the presented interpretation of luminescence Nd^3+^ spectra for a quantitative estimation *f*_a_ in orthophosphates is, in principle, applicable to other REE^3+^ probes, that may be substituted in alternative nuclear waste-form materials and their mineral analogs, e.g., apatite Ca_5_(PO_4_)_3_(F,Cl,OH), zircon ZrSiO_4_, titanite CaTiSiO_5_, zirconolite CaZrTi_2_O_7_.

## Author Contributions

All authors listed have made a substantial, direct, and intellectual contribution to the work. CL and GL initiated and led the study. Sample synthesis was done by DG and CL. Ion irradiations were performed by MI. Electron microscopy (including SEM and TEM analysis) and FIB preparation are conducted by RA and JD. X-Ray diffraction analysis were performed by GT. The manuscript was compiled and prepared by CL.

### Conflict of Interest Statement

The authors declare that the research was conducted in the absence of any commercial or financial relationships that could be construed as a potential conflict of interest.
